# Highly efficient gene editing and single cell analysis of hematopoietic stem/progenitor cells from X-linked sideroblastic anemia patients

**DOI:** 10.1038/s41392-021-00622-3

**Published:** 2021-07-02

**Authors:** Riguo Fang, Jingliao Zhang, Huihui Yang, Jia Shi, Huimin Zeng, Xiaofan Zhu, Dong Wei, Pengfei Yuan, Tao Cheng, Yingchi Zhang

**Affiliations:** 1 EdiGene Inc., Beijing, China; 2 EdiGene (Guangzhou) Inc., Guangzhou, China; 3 State Key Laboratory of Experimental Hematology, National Clinical Research Center for Blood Diseases, Institute of Hematology & Blood Diseases Hospital, Chinese Academy of Medical Sciences & Peking Union Medical College, Tianjin, China; 4 Department of Pediatrics, Peking University People’s Hospital, Beijing, China

**Keywords:** Haematopoietic stem cells, Gene therapy

**Dear Editor**,

X-linked sideroblastic anemia (XLSA), which is the most common genetic form of congenital sideroblastic anemia, is typically characterized by reduced heme synthesis and the presence of bone marrow (BM) ring sideroblasts containing pathologic iron deposits in the mitochondria.^1^ Notably, most of the reported XLSA male cases are caused by mutations in the gene encoding 5-aminolevulinate synthase 2 (ALAS2). Hematopoietic stem cells (HSCs) transplantation is the only effective treatment to cure XLSA, but with limitations including the uncertain availability of suitable donors and transplantation-associated mortality and morbidity such as graft-versus-host disease and immune rejection. Clustered regularly interspaced short palindromic repeats-associated protein-9 nuclease (CRISPR/Cas9) technology, has shown tremendous potential for the clinical treatment of inherited diseases through transplantation of genetically modified HSCs.^2^ Here, we developed an efficient gene-editing platform to repair pathogenic mutations in the ALAS2 gene in CD34^+^ hematopoietic stem and progenitor cells (HSPCs) from XLSA patients, providing the groundwork for a potential therapy for XLSA (Supplemental Fig. S1).

Previously, we and others have identified the A>G mutation ([Chr X (GRCh37/hg19): g.55054635A>G]) in the GATA1 binding region of ALAS2 intron 1 in particular XLSA families^3^ (Supplemental Fig. S2), and have demonstrated the key role of this site in regulating ALAS expression. Hence, we firstly designed a series of synthetic guide RNAs (sgRNAs), along with single-stranded DNA oligonucleotide donors (ssODN) (Fig. 1a), which were co-electroporated with Cas9 mRNA into hiPSCs derived from XLSA patients. After optimization, next generation sequencing analysis (NGS) showed that a maximum homology-directed repair (HDR) rates up to 26.3% were achieved (Supplemental Fig. S3). Following our demonstration of genetic correction at ALAS2 disease mutation in hiPSCs, we next evaluated our gene-editing strategy in CD34^+^ HSPCs from two patients (X037 and X041) in the XLSA pedigrees. We achieved high levels of gene correction (HDR), with an average of 43.93 ± 3.43% (Fig. 1b and Supplemental Fig. S4). To examine the potential for reversal of the XLSA phenotype by gene correction, we induced erythroid differentiation of CD34^+^ HSPCs. Analysis of the number of CD71^+^ and CD235^+^ erythroblasts revealed partial recovery of erythroid differentiation potential in gene-corrected cells compared with healthy donor and mock-treated cells (Supplemental Fig. S5a). Surprisingly, benzidine staining also showed that gene-correction significantly increased the heme biosynthesis compared with the mock-treated and healthy donor cells (Fig. 1c, d). Moreover, *ALAS2* mRNA and protein levels in gene-edited cells were markedly increased to nearly 50% of those in healthy donor cells, while *GATA1* expression was almost unchanged (Fig. 1e and Supplemental Fig. S5b).

**Fig. 1:**
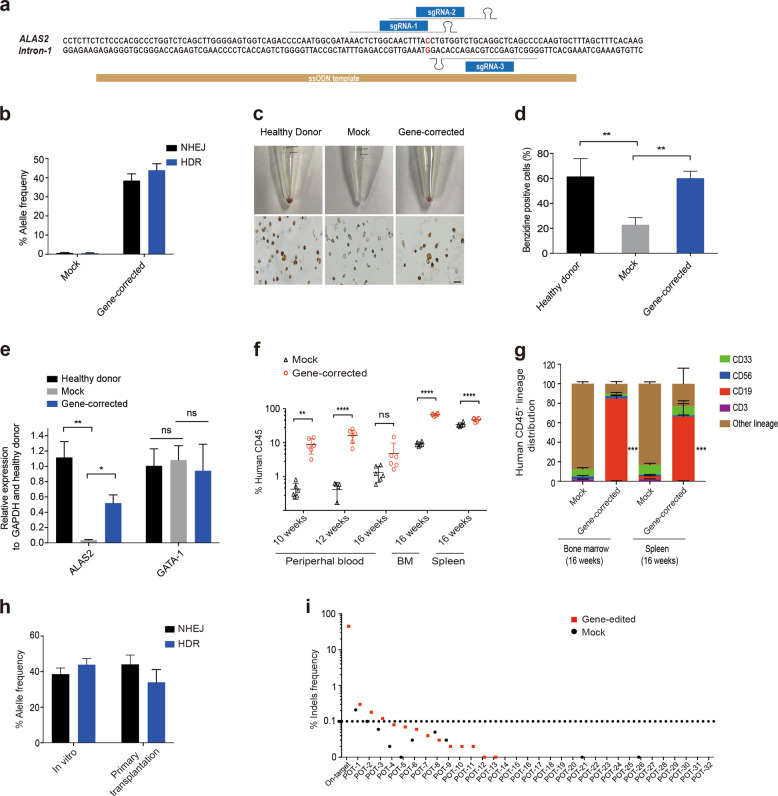
ALAS2 gene correction in CD34^+^ HSPCs from XLSA patients. **a** Schematic representation of sgRNAs and ssODN template designs near the mutations in intron 1 of *ALAS2* (uppercase, red). **b** *ALAS2* gene correction in CD34^+^ HSPCs from XLSA patients using Cas9 mRNA, sgRNA-1 and ssODN. The allele frequencies of HDR and NHEJ were detected by NGS analysis. Mock: unedited CD34^+^ HSPCs from XLSA patients. **c** Erythroid cell pellets, benzidine staining after 18 days of differentiation. Scale bar = 20 μm. **d** The percentage of benzidine-positive cells. **e** *ALAS2* and *GATA1* mRNA expression in erythroid cells was analyzed by RT-qPCR after 18 days of differentiation, and the results are normalized to those in healthy donor cells. GAPDH served as the internal control. Mock and gene-corrected indicate erythroid cells derived from unedited and edited CD34^+^ HSPCs from XLSA patients, respectively. Healthy donor indicates normal CD34^+^ HSPCs from granulocyte colony-stimulating factor (G-CSF)-mobilized CD34^+^ HSPCs. **f** Human CD45^+^ cell reconstitution was evaluated in the peripheral blood, bone marrow, and spleen of NPG mice transplanted with gene-corrected CD34^+^ HSPCs; unedited cells were transplanted as the mock control. *n* = 6 mice per group. **g** Lineage distribution of human CD45^+^ cells in the bone marrow and spleen of primary recipient mice 16 weeks after transplantation. **h** Gene correction rate in bulk transplanted cells at the time of engraftment (in vitro) and in bone marrow cells at 16 weeks post transplantation. The HDR and NHEJ rates were evaluated by NGS analysis. **i** Potential off-target sites were identified by in silico prediction and unbiased Digenome-seq and then directly interrogated in hiPSCs by targeted PCR amplification and NGS analysis. Off-target sites were plotted on the *X* axis grouped as an on-target site or potential off-target (POT) site and then sorted by the mean indel percentage. The number of indels detected by NGS using ampliCan is plotted on the *Y* axis on a log scale. All in vitro experiments were performed independently in triplicate. All data are shown as the mean ± SD. *P < 0.05, **P < 0.01, ***P < 0.001, ****P < 0.0001

To evaluate the multilineage differentiation potential after gene-editing, we first performed colony-forming unit (CFU) assays. Compared with mock-treated cells, gene-editing significantly enhanced the generation of total, CFU-GM (CFU- granulocyte/macrophage) and BFU-E (burst-forming unit-erythroid) colonies, suggesting the higher clonogenic potential of these cells (Supplemental Fig. S6). Next, to assess the in vivo repopulating potential, gene-corrected cells were transplanted into nonobese diabetic (NOD)/*Prkdc*^scid^/IL-2Rγ^null^ (NPG) mice. All transplanted mice of gene-corrected group displayed engraftment in multiple organs at 10–16 weeks post-transplantation, suggesting greater engraftment capacity than the mock group, as measured by human CD45 of total CD45 (Fig. 1f and Supplemental Fig. S7a). Furthermore, hematopoietic reconstitution analysis indicated that the gene-corrected cells maintained different lineage distribution (Fig. 1g and Supplemental Fig. S7b). Next, gene-editing efficiency analysis of bone marrow samples 16 weeks after transplantation exhibited high editing rate (34 ± 7.18% via HDR), comparable to the in vitro efficiency (Fig. 1h). To further investigate the long-term reconstitution capacity, we performed secondary transplantation assay and observed engraftment in the BM for gene-corrected cells but not mock group 12 weeks after transplantation, while the HDR rate was 42.9% on average, suggesting long-term sustained gene editing effects (Supplemental Fig. S7c, d). Furthermore, to determine the specificity of our gene editing system we used unbiased Digenome-seq to identify potential off-target sites and deeply interrogated via targeted PCR and NGS analysis of XLSA hiPSCs treated with Cas9 mRNA and sgRNA. No off-target cleavage events were detected at these sites, indicating a lack of detectable off-target events (Fig. 1i).

CD34^+^ HSPCs were collected for gene-editing from two patients in the XLSA pedigree. Only the CD34^+^ HSPCs from the younger patient were successfully gene-corrected (data not shown). Moreover, older XLSA patients showed weak hematopoiesis, indicating that HSC function might be affected by increased age. Thus, we used scRNA-seq technology to analyze the composition and transcriptional characteristics of HSPCs from XLSA patients of different ages. In this XLSA pedigree, CD34^+^ HSPCs from three XLSA patients (X037, aged 26 years, X039, aged 15 years, and X041 aged 4 years), were investigated by scRNA-seq, with CD34^+^ HSPCs from three healthy donors (HD) serving as the normal control. Droplet-based 5′scRNA-seq libraries were generated from three HD and three XLSA patients. A total 48,669 high-quality single-cell profiles from these six donors were obtained. We then identified 11 cell clusters based on single cell profiles and marker gene expression (Supplemental Fig. S8a). Eleven clusters were identified in the investigated donors, although the percentage of each cluster was different (Supplemental Fig. S8b). Notably, the percentages of HSC, multi-lineage progenitor cell (MultiLin) and erythroid progenitor cell (ERP) in oldest XLSA patient(X037) were higher than these in HD and younger patients (YP) (Supplemental Fig. S8c). In addition, we observed an increased proportion of G1 phase cells and a decreased proportion of G2/M phase cells among the HSCs from patient X037, compared to those from HD and YP (Supplemental Fig. S8d). These results suggested that HSCs from patient X037 were more inactive as for cell cycle status, which may contribute to the relatively lower editing efficiency of the HSCs from patient X037. Single cell gene expression profiling further revealed that the transcriptome characteristics of HSC in oldest patient X037, were significantly different from that in HD and YP (X041, X039) (Supplemental Fig. S8e). JUN and FOS, two components of transcription factor AP-1 participating in lots of biological processes such as proliferation, differentiation, apoptosis and development,^4^ significantly increased in HSC of XLSA patients compared with HD. Moreover, the percentage of FOS and JUN high-expression cells in oldest patient X037 was more than that in YP (Supplemental Fig. S8f). It was reported that prolonged expression of c-fos kept more mouse HSPCs into G0/G1 phase of cell cycle,^5^ and this may be the reason why more HSCs from oldest patient X037 were in G1 phase of cycle. Additionally, HOPX and SPINK2, two predictors of poor prognosis of acute myeloid leukemia (AML), were also increased in HSC of oldest patient X037. These data indicate that older XLSA patients have a higher proportion of HSCs that are arrested in the G1 phase of the cell cycle, which may be associated with a higher probability of gene-editing failure and abnormal hematopoiesis.

In conclusion, we have demonstrated for the first time the use of non-viral, selection-free gene-editing strategy based on CRISPR/Cas9 technology to correct the disease mutation and rescue ALAS2 expression and heme biosynthesis. This study not only provides direct evidence of the ALAS2 mutation as a causative factor in the pathogenesis of human XLSA, but also a comprehensively transcriptional profile of CD34^+^ HSPCs from XLSA patients at single-cell resolution for the first time. Thus, our findings provide an important insights into the mechanism underlying the pathogenesis of XLSA.

## Supplementary information

SUPPLEMENTAL MATERIAL
