# Supplementary material for: Highly efficient gene editing and single cell analysis of hematopoietic stem/progenitor cells from X-linked sideroblastic anemia patients
Source: Signal Transduct Target Ther. 2021 Jul 2;6:248. doi: 10.1038/s41392-021-00622-3 (PMC8249499; doi:10.1038/s41392-021-00622-3)
Supplement: Supplementary file 1 — SUPPLEMENTAL MATERIAL [file 41392_2021_622_MOESM1_ESM.docx]

Supplementary Materials for

Highly Efficient Gene Editing and Single Cell Analysis of Hematopoietic Stem/Progenitor Cells from X-linked Sideroblastic Anemia Patients

Riguo Fang^1,4^, Jingliao Zhang^2,4^, Huihui Yang^1^, Jia Shi^1^, Huimin Zeng^3^, Xiaofan Zhu^2^, Dong Wei^1^, Pengfei Yuan^1,5^, Tao Cheng^2,5^, Yingchi Zhang^2,5^

^1^EdiGene Inc., Beijing, China. EdiGene (Guangzhou) Inc., Guangzhou, China.

^2^State Key Laboratory of Experimental Hematology, National Clinical Research Center for Blood Diseases, Institute of Hematology & Blood Diseases Hospital, Chinese Academy of Medical Sciences & Peking Union Medical College, Tianjin, China.

^3^Department of Pediatrics Peking University People's Hospital.

^4^These authors contributed equally: Riguo Fang and Jingliao Zhang.

^5^Corresponding authors: Yingchi Zhang, Tao Cheng, Pengfei Yuan.

Correspondence to: [zhangyingchi@ihcams.ac.cn](mailto:zhangyingchi@ihcams.ac.cn); [chengtao@ihcams.ac.cn](mailto:chengtao@ihcams.ac.cn); pfyuan@edigene.com;

**This PDF file includes:**

Materials and Methods

Supplementary Text

Figures. S1 to S8

Tables S1 to S4

Materials and Methods

**Isolation of human CD34^+^ HSPCs**

Bone marrow and granulocyte colony-stimulating factor (G-CSF)-mobilized peripheral blood samples were obtained as donated samples with approval from the Institutional Review Board of the Institute of Hematology, Blood Diseases Hospital, Peking Union Medical College/Chinese Academy of Medical Sciences (PUMC/CAMS). Mononuclear cells were isolated from cell suspensions using Ficoll (Dakewe). Human CD34^+^ hematopoietic stem and progenitor cells (HSPCs) were purified by microbeads conjugated with anti-human CD34 antibodies (Miltenyi Biotec) and a magnetic-activated cell sorting system (Miltenyi Biotec) according to the manufacturer’s instructions.

**Generation of human iPSCs from XLSA patient peripheral blood samples**

We established XLSA patient-derived human iPSCs using a previously reported optimized protocol^1^. Briefly, peripheral blood mononuclear cells (PB-MNCs) were cultured in StemSpan serum-free expansion medium II (SFEM II, STEMCELL Technologies) supplemented with erythroid expansion supplement (STEMCELL Technologies), stem cell factor (SCF, 100 ng/mL, PeproTech), interleukin-3 (IL-3, 10 ng/ml, PeproTech), erythropoietin (EPO, 2 U/ml, PeproTech), insulin growth factor-1 (IGF-1, 20 ng/ml, PeproTech), dexamethasone (1 μM, Sigma) and 1-thioglycerol (0.2 mM, Sigma). After cell culture for 6 days, 2x10^6^ cells were electroporated with episomal reprogramming vectors, and 5x10^4^-1x10^6^ of cells were plated on gelatin-treated plates with mitomycin-inactivated murine embryonic fibroblast feeder cells. After 14 days, hiPSCs were manually picked, cultured in vitronectin-precoated-well plates and refreshed with Essential 8^TM^ medium (ThermoFisher Scientific) daily for long-term propagation.

**Cell culture**

Human CD34^+^ HSPCs were cultured in SFEM II (STEMCELL Technologies) supplemented with SCF (100 ng/mL, PeproTech), fms-related tyrosine kinase 3 ligand (Flt-3l, 100 ng/mL, PeproTech), and thrombopoietin (TPO, 100 ng/mL, PeproTech). hiPSCs were cultured and propagated in a xeno-free and feeder-free system using Essential 8TM medium (ThermoFisher Scientific) and vitronectin (ThermoFisher Scientific). CD34^+^ HSPCs and hiPSCs were cultured at 37°C with 5% CO_2_ in a humidified incubator.

**Electroporation**

XLSA patient-derived hiPSCs were cultured in Essential 8TM medium. A total of 1 to 2x10^6^ hiPSCs were nucleofected using a 4D nucleofector device (Lonza) with different amounts of Cas9 mRNA, sgRNA, and ssODN template, and replated in vitronectin-precoated well plates and cultured in Essential 8TM medium (ThermoFisher Scientific) daily. The nucleofected cells were cultured for 3 days at 37°C with 5% CO_2_ in a humidified incubator for further analysis.

CD34^+^ HSPCs were prestimulated for 24 to 48 hours in culture medium. A total of 2x10^5^ CD34^+^ cells were then electroporated in 100 μl BTX press buffer with a BTX AgilePulse Electroporator (250 V, 0.5 ms, 1 pulse) or in 100 μl P3 solution with the Lonza 4D nucleofector device (Program ER100) according to the manufacturers’ instructions. Gene editing solutions contained 6 μg Cas9 mRNA and 4 μg sgRNA. To correct the point mutation in CD34^+^ HSPCs from XLSA patients, 6 μg Cas9 mRNA, 4 μg sgRNA and 12 μg ssODN template were co-delivered into 2x10^5^ CD34^+^ cells using the BTX ECM 830 system. For mock-treated CD34^+^ cells, the same number of cells was electroporated using the BTX ECM 830 system, but the CRISPR components and ssODN were not included. The electroporated cells were cultured for 2 days at 37°C with 5% CO_2_ in a humidified incubator for further analysis.

**Analysis of genome editing**

Genomic DNA was extracted from each sample using the QIAamp DNA Mini Kit (Qiagen) according to the manufacturer’s protocol. Then, regions containing targeted and potential off-target sites (~250 bp) were amplified using PCR with primers listed in the supplemental table. For ICE analysis, the PCR product was sequenced and then analyzed using ICE (https://ice.synthego.com/#/). For NGS analysis, DNA libraries were prepared with the NEBNext® UltraTM DNA Library Prep Kit (Illumina) and then analyzed on the Illumina X-ten platform. Low-quality data were filtered out, and clean data were merged using vsearch. Filtered sequences were then aligned to the expected sequence in blastn. To calculate the indel efficiency, all possible deletions (based on the start site and length) fully within ± 25 bp of the cut site and with at least 1 bp within ± 5 bp of the cut site were considered. For the gene correction efficiency, the G/A rate in sequences without any indels was calculated.

**Erythroid differentiation**

For the erythroid differentiation of CD34^+^ HSPCs from XLSA patients, we used a previously reported two-step differentiation protocol^2^. Briefly, 2 days after electroporation, CD34^+^ HSPCs were cultured for 7 days at an optimal density (0.5 to 2x10^5^/ml) in step 1 medium consisting of SFME II and StemSpan™ Erythroid Expansion Supplement (STEMCELL Technologies). On day 8 (step 2), the resulting erythroid progenitors were transferred to SFME II supplemented with 3 U/ml erythropoietin (STEMCELL Technologies), 3% human AB serum (Sigma Aldrich) and 1 μM mifepristone (Sigma) at an optimal density (0.5 to 2x10^6^/ml). After 7-10 days, mature erythroid cells were collected to assess the differentiation efficiency by flow cytometry analysis, staining for CD71 (BioLegend, 334108) and CD235a (BioLegend, 349106), and other further experiments.

**CFU assay**

48 hours after electroporation, human CD34^+^ HSPCs (500 cells) suspended in 100 μl of Iscove’s modified Dulbecco’s medium supplemented with 2% FBS (STEMCELL Technologies) were mixed with 1 ml of methylcellulose (MethoCult™ H4034 Optimum, STEMCELL Technologies). After plating on a SmartDishTM 6-well plate using a 3 cc syringe and blunt-end needle (STEMCELL Technologies), the cells were cultured for 14 days at 37°C in a 5% CO_2_ humidified incubator. Colonies derived from healthy donor cells, gene-corrected cells and untreated-mock cells were counted and scored based on morphological features.

**Transplantation and engraftment assay**

NOD/Prkdcscid/IL-2Rγnull (NPG) mice were purchased from Beijing Vitalstar Biotechnology. Within 24 hours, 5- to 6-week-old NPG mice were sub-lethally irradiated (1.6 Gy) and transplanted with 1 million unedited or gene-corrected CD34^+^ HSPCs resuspended in 120 μl of freshly prepared CD34^+^ HSPC culture media. For secondary transplantation, bone marrow cells harvested from one 16-week-old reconstituted mouse were injected into two 5- to 6-week-old sub-lethally irradiated (1.6 Gy) NPG mice.

Mouse peripheral blood samples were harvested every 2 weeks beginning 10 weeks post transplantation. Human cell engraftment and multilineage differentiation potential were determined by flow cytometry analysis using the following BioLegend antibodies: human CD45 (APC/Cy7. 304014), human CD3 (PerCP, 300428), human CD19 (PE, 363004), human CD33 (BV421, 303416), human CD56 (APC, 304610), and mouse CD45 (FITC, 103108). Genomic DNA was extracted from the bone marrow of 16-week-old transplanted mice and 12-week-old secondary transplanted mice and subjected to editing rate analysis to quantify the NHEJ and HDR rates as described above.

**Western blot analysis**

Western blot analysis was performed as previously described^3^. Briefly, cells were lysed in Laemmli sample buffer (BioRad, 161-0737), and lysates were separated by sodium dodecyl sulfate polyacrylamide gel electrophoresis (SDS-PAGE). Proteins were transferred to a nitrocellulose membrane, which was incubated with antibodies against GAPDH (Cell Signaling Technology, 5174), ALAS2 (Abcam, ab184964) and GATA1 (Abcam, ab11852). Horseradish peroxidase-conjugated secondary antibodies were used. Signals were detected using an ECL kit (ThermoFisher Scientific, WP20005).

**RNA extraction and qRT-PCR**

Total RNA was extracted from cells using the RNeasy Plus Micro Kit (QIAGEN), and cDNA was synthesized using TransScript One-Step gDNA Removal and cDNA Synthesis SuperMix (TransGen Biotech). Quantitative real-time PCR (qRT-PCR) was performed using the FastStart Essential DNA Green Master Kit (Roche, 06402712001). Primers are listed in supplemental Table S3.

**Off-target analysis**

Potential off-target sites were predicted using unbiased Digenome-seq. Briefly, genomic DNA was extracted from 10^6^ hiPSCs from XLSA patients. The ALAS2 target region was amplified using PCR with the primers listed in the supplemental table, and this product served as a positive control. Both genomic DNA and the amplicon were then incubated with Cas9 protein (NEB M0386) and ALAS2 sgRNA (Synthego) for 2 h at 37°C. After incubation, the amplicon was subjected to agarose electrophoresis to confirm complete cleavage. Then, the genomic DNA was subjected to whole genome sequencing. Data analysis was performed as previously described^4,5^. The primers used to analyze the potential off-target were listed in Table S4.

**Sample preparation for scRNA-seq**

The human samples were collected from 3 healthy children donors and 3 diagnosed XLSA children from the pedigree (Fig. S1). The Ethics Committee of Tianjin Blood Disease Hospital approved the study protocol, both the donor and patients consented to sample collection and data analysis. Density gradient centrifugation were used to isolate bone marrow mononuclear cells (BMMCs) from the iliac crest aspirates of healthy donors and XLSA patients. The isolated bone marrow mononuclear cells were viably frozen in fetal bovine serum (FBS) (Gibco，12483020) with 10% dimethyl sulfoxide (DMSO) (Sigma Aldrich, D5879), then cryopreserved in liquid nitrogen until further use. Frozen cells were thawed quickly by swirling it in a 37 °C water bath, then viable cells were transferred into a sterile 5ml round bottom tube with digestion buffer containing 0.1mg/ml DNase I (Roche, 11284932001) and 10% FBS in IMDM medium (Gibco, 12440053). After being incubated for 15 minutes at room temperature，the tubes were centrifuged at 300g for 10 minutes at 4 °C temperature to collect the cells, then the supernatant was removed and discarded, and the cells were resuspended in 100ul sorting buffer containing DPBS (Gibco, 14190144) with 2% FBS, 1% Penicillin-Streptomycin (Gibco, 15140122) and 2mmol/l EDTA (Thermo Fisher Scientific, 17892). Next, samples were stained with APC-conjugated anti-human CD34 antibody (BD, 555824) as needed at 4 °C temperature for 30 minutes. After that, cells were washed with 1ml sorting buffer, and then passed through a 70-um cell strainer. DAPI (Sigma-Aldrich, D9542) was added to cells prior to fluorescence activated cell sorting (FACS) using an Aria III flow cytometer with four lasers (375, 488, 561and 633 nm) (BD, 648282). Live cells were sorted into a sterile 5ml round bottom tube containing 3ml cold DPBS with 0.1%BSA.

**Single-cell library preparation and sequencing**

Cell suspensions were evaluated for viability and concentration with filtered trypan blue by automated cell counter (Bio-Rad, 145-0102). Optimal range of cell stock concentration is between 700 to 1200 cells per microliter to maximize the likelihood of achieving the desired cell recovery target, and samples’ viabilities should be ≥85% to improve the recovery rate. According to the results of cell counter and recommended cell concentration, required number of cells were resuspended by certain volume of cold DPBS with 0.04% BSA. Single cells were captured and barcoded in 10x Chromium controller (10x Genomics). The CD34^+^ cells from bone marrow were captured in multi-chip with separated channels. Subsequently, single-cell RNA-Seq libraries were prepared using Chromium Single Cell 3’v3 Reagent Kit (10x Genomics). Sequencing libraries were loaded on an Illumina NovaSeq with 2×150 paired-end kits at Novogene.

**Pre-processing Single-cell RNA-seq data**

The FASTQ files were analyzed with the Cell Ranger Software Suite (version 3.1.0;10x Genomics). We compared the raw data with human genome reference (GRCh38) to generate the filtered gene-barcode matrix which contained valid cell barcodes and transcript UMI counts with Cell Ranger Count.

**scRNA-seq Data Analysis with Seurat**

The filtered gene-barcode matrix of each sample identified by CellRanger Count was inputted into Seurat (version 3.1.0)^6^. To further filter cells and genes in Seurat, we removed cell outliers (retain cells with more than 200 genes), cells with high mitochondrial transcript proportion (retain cells with mito% less than 5~30%, judging by 99% fractile),cells with high hemoglobin level(more than 5%),as well as genes which were detected in less than three cells. Then, we normalized the filtered gene-barcode matrix by total expression of each sample. To avoid biasing to donor and batch differences, we used Harmony^7^ for integrated analysis of six datasets. “FindVariableFeatures” in Seurat was applied to select highly variable genes. Using “FindClusters” function for clustering we obtained unique clusters and “FindAllMarkers” was designed to identify lineage-specific markers. The clusters were visualized with UMAP plots with resolution set 0.5 as implemented in Seurat.Cell cycle phase scores was evaluated by the “CellCycleScoring” function based on canonical markers in Seurat, along with the predicted classification of each cell in either G2M, S or G1 phase.

**Annotation of Clusters**

Manual annotation. Annotation of clusters to cell types was done by manual inspection of the genes defining each cluster and comparison to the literature. To identify genes that were most important in defining each cluster we performed differential expression analysis for genes enriched within a cluster relative to all other clusters (e.g., cluster 1 versus all cells not in cluster 1) by a hypergeometric test with a 0.05 p-value cut-off after multiple hypothesis correction.

An automatic tool. We developed an algorithm referred to SCSA^8^, which can automatically assign cell types for each cell cluster in scRNA-seq data. First, the input were differentially expressed genes as marker genes which were output results of Seurat. Next, for each cluster, the algorithm used a decision model to assign an enrichment score estimating the strength of evidence for marker genes in the human bone marrow single-cell atlas^9^ by hypergeometric distribution test.

**Differential Expression Analysis**

Differentially expressed genes were analyzed using edgeR^10^ across cell types to identify cluster-specific genes or across healthy donors and different patients of the same cell type. Differential gene expression was calculated using UMI normalized gene expression data by t-test with multiple hypothesis correction (Benjamini-Hochberg correction). Genes encoding ribosomal proteins were excluded. The significantly differential expression genes with stringent threshold value (t-test, adjusted p-value/FDR<0.05) were further investigated with the gene enrichment analysis tool clusterProfiler R package based on GO, KEGG and Reactome libraries.

**Statistical analysis**

Student’s t test was used for comparisons between two groups, and two-way ANOVA was used for comparisons among multiple groups. P values of <0.05 were considered statistically significant. *p<0.05; **p<0.01; ***p<0.001; ****p<0.0001; ns, not significant.

**Data availability**

The datasets used and/or analyzed during the current study are available from the corresponding author on reasonable request.

**Reference**

1 Wen, W. *et al.* Enhanced Generation of Integration-free iPSCs from Human Adult Peripheral Blood Mononuclear Cells with an Optimal Combination of Episomal Vectors. *Stem Cell Reports* **6**, 873-884, doi:10.1016/j.stemcr.2016.04.005 (2016).

2 DeWitt, M. A. *et al.* Selection-free genome editing of the sickle mutation in human adult hematopoietic stem/progenitor cells. *Sci Transl Med* **8**, 360ra134, doi:10.1126/scitranslmed.aaf9336 (2016).

3 Liu, J. *et al.* Long non-coding RNA-dependent mechanism to regulate heme biosynthesis and erythrocyte development. *Nat Commun* **9**, 4386, doi:10.1038/s41467-018-06883-x (2018).

4 Kim, D. *et al.* Digenome-seq: genome-wide profiling of CRISPR-Cas9 off-target effects in human cells. *Nat Methods* **12**, 237-243, 231 p following 243, doi:10.1038/nmeth.3284 (2015).

5 Kim, D. & Kim, J. S. DIG-seq: a genome-wide CRISPR off-target profiling method using chromatin DNA. *Genome Res* **28**, 1894-1900, doi:10.1101/gr.236620.118 (2018).

6 Butler, A., Hoffman, P., Smibert, P., Papalexi, E. & Satija, R. Integrating single-cell transcriptomic data across different conditions, technologies, and species. *Nat Biotechnol* **36**, 411-420, doi:10.1038/nbt.4096 (2018).

7 Magella, B. *et al.* Cross-platform single cell analysis of kidney development shows stromal cells express Gdnf. *Dev Biol* **434**, 36-47, doi:10.1016/j.ydbio.2017.11.006 (2018).

8 Cao, Y., Wang, X. & Peng, G. SCSA: A Cell Type Annotation Tool for Single-Cell RNA-seq Data. *Front Genet* **11**, 490, doi:10.3389/fgene.2020.00490 (2020).

9 Hay, S. B., Ferchen, K., Chetal, K., Grimes, H. L. & Salomonis, N. The Human Cell Atlas bone marrow single-cell interactive web portal. *Exp Hematol* **68**, 51-61, doi:10.1016/j.exphem.2018.09.004 (2018).

10 Robinson, M. D., McCarthy, D. J. & Smyth, G. K. edgeR: a Bioconductor package for differential expression analysis of digital gene expression data. *Bioinformatics* **26**, 139-140, doi:10.1093/bioinformatics/btp616 (2010).

Figure. S1. The flowchart of the research ideas in this study. Firstly, we established XLSA patient’s specific iPSCs and performed a series of experiment optimization to select the optimal combination of Cas9 mRNA, sgRNA and ssODN. Secondly, following successful gene correction of pathogenic gene mutation on hiPSCs, we high efficiently repaired the disease mutation on CD34^+^ HSPCs from patients with XLSA. In vitro erythroid differentiation analysis demonstrated that gene-correction repaired the function of ALAS2 gene mutation and in vivo animal study showed the repaired capabilities of engraftment and hematopoietic reconstitution. Finally, to investigate whether only the CD34^+^ HSPCs from the younger patient rather than older patient were successfully gene-corrected, we performed the single cell RNA-sequencing analysis of CD34^+^ HSPCs from patients with XLSA and healthy donors.

**
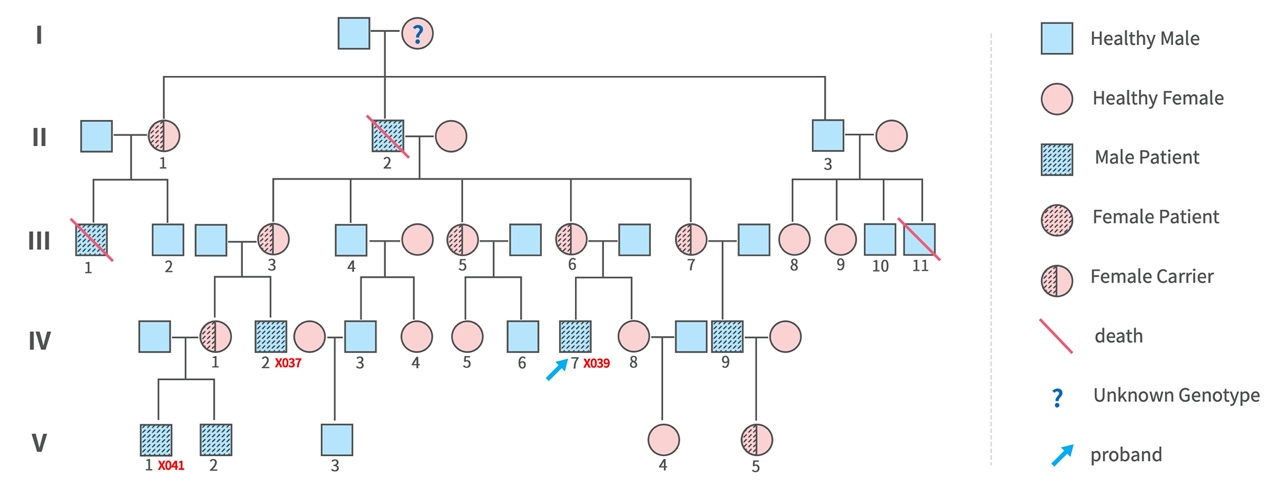
**

**Figure. S2. The flowchart of the research ideas in this study.** The blue shaded boxes indicate the affected individuals in this pedigree. The blue arrow indicates the proband in this XLSA family. The CD34^+^ HSPCs of patient IV2(#X037) and V1(#X041) were used in gene-editing study, while the CD34^+^ HSPCs of patient IV2(#X037), IV7(#X039) and V1(#X041) were used in scRNA-seq study.

**
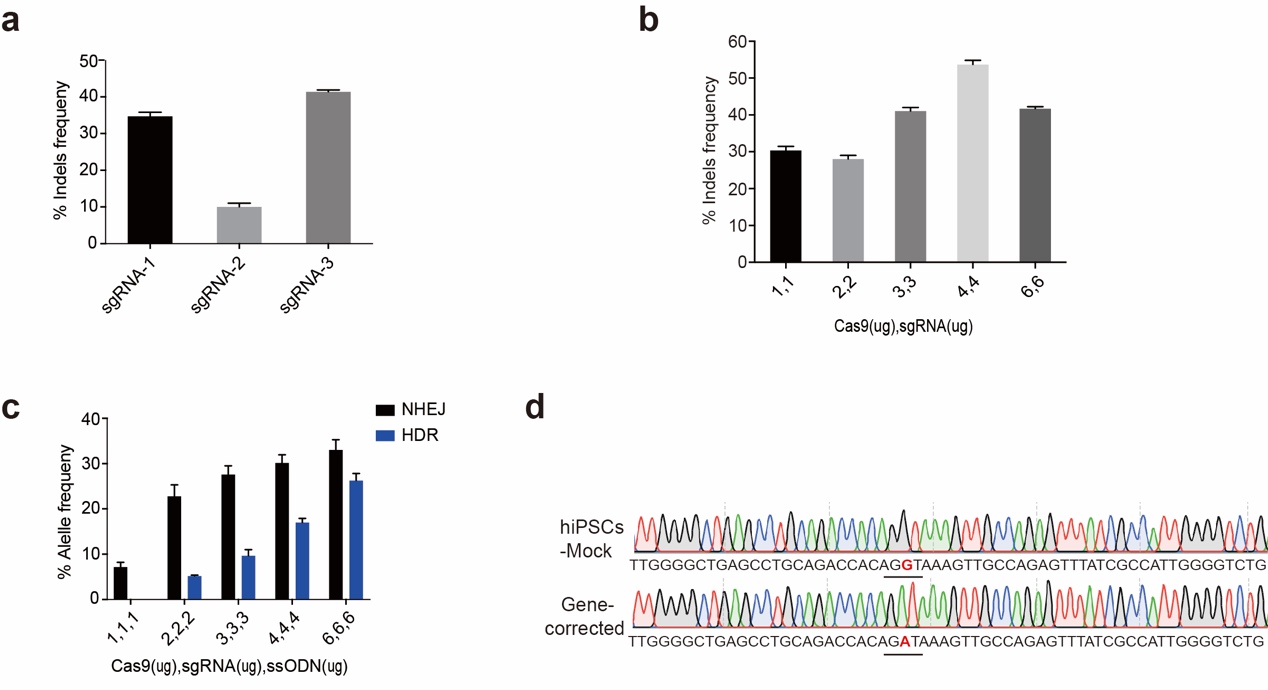
**

**Figure. S3. Correction of ALAS2 gene mutations in hiPSCs derived from XLSA patients**. (a) The percentage of indels after the electroporation of ALAS2 sgRNA1-3 and Cas9 mRNA into XLSA patient-derived hiPSCs was quantified by Sanger sequencing and the ICE CRISPR Analysis Tool (SYNTHEGO). (b) Indels rates after the introduction of different amounts of Cas9 mRNA and sgRNA-1 into XLSA patient-derived hiPSCs, as detected by NGS analysis. n=3 independent experiments. (c) The allele frequencies of gene correction (HDR) and Indels (NHEJ) after the delivery of different amounts of Cas9 mRNA, sgRNA-1, and ssODN into XLSA patient-derived hiPSCs were measured by NGS analysis. (d) Representative Sanger sequencing analysis of the G>A correction (red, uppercase) in hiPSCs from XLSA patients before and after gene editing. The reference sequence is GRCh37/hg19ChrX: 55054615–55054661. All data are shown as the mean±SD.

**
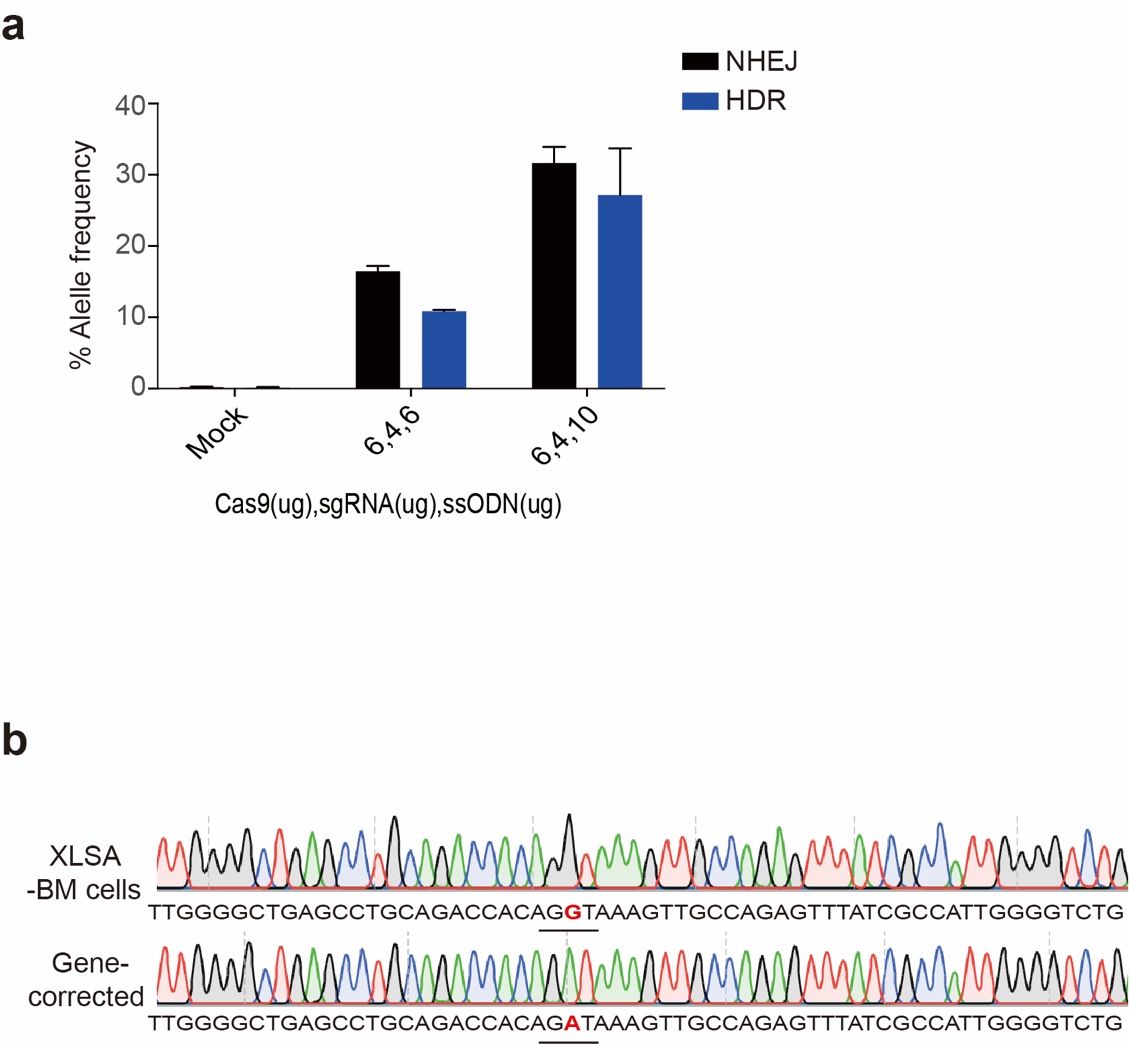
**

**Figure. S4. Correction of ALAS2 gene mutations in CD34^+^ HSPCs derived from XLSA patients bone marrow (BM)**. (a) The allele frequencies of gene correction (HDR) and Indels (NHEJ) after the introduction of different amounts of Cas9 mRNA, sgRNA-1, and ssODN into XLSA patient-derived CD34^+^ HSPCs were measured by NGS analysis. n=3 independent experiments. (b) Representative Sanger sequencing data of the G>A correction in XLSA BM cells and CD34^+^ HSPCs after gene editing. The reference sequence is GRCh37/hg19ChrX: 55054615–55054661. All data are shown as the mean±SD.


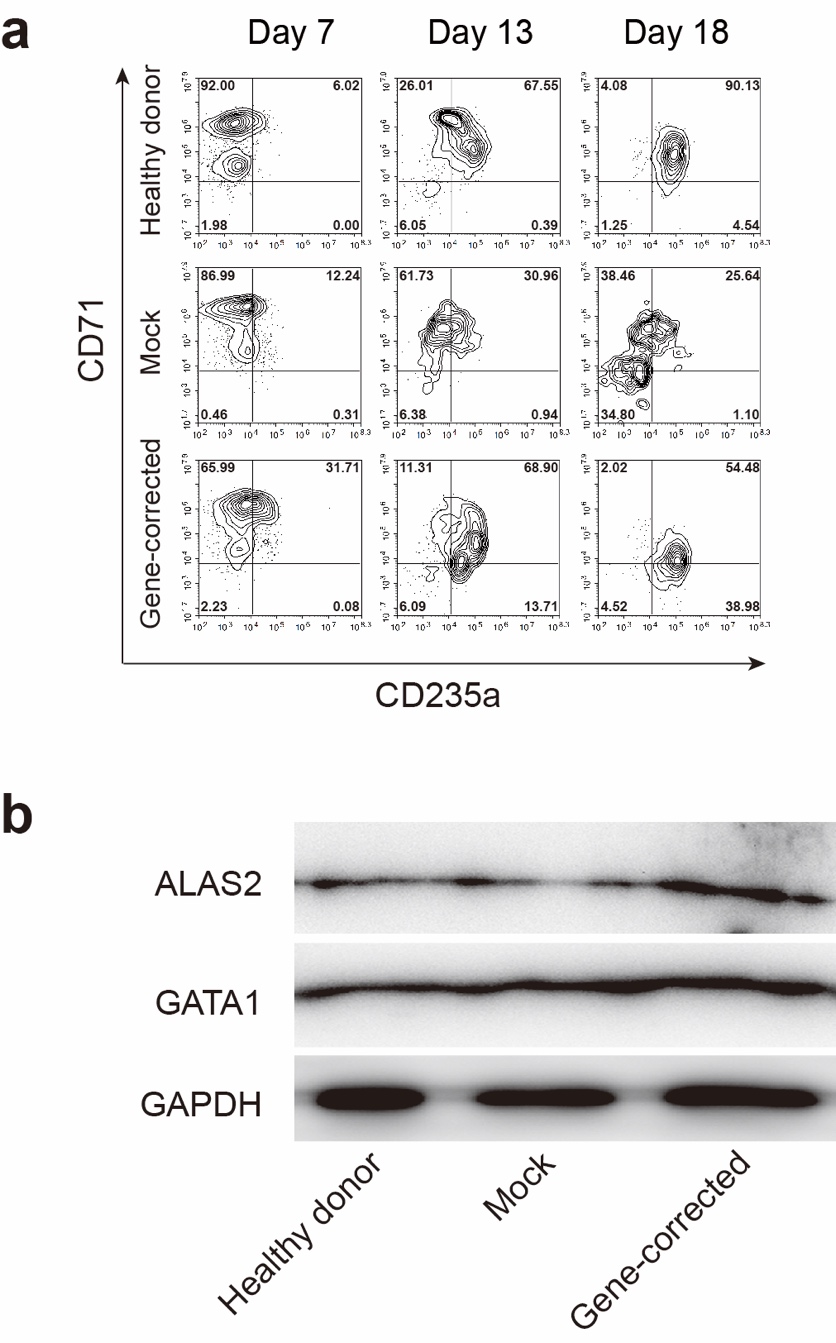


**Figure. S5. Erythroid differentiation and western blot analysis after ALAS2 gene correction.** (a) Erythroid differentiation of CD34^+^ HSPCs was induced ex vivo and monitored by flow cytometry analysis (staining with anti-CD71 and anti-CD235a antibodies). Mock: erythroid cells derived from unedited CD34^+^ HSPCs from XLSA patients. (b) ALAS2 and GATA1 protein levels were measured by western blotting. Healthy donor: erythroid cells differentiated from CD34^+^ HSPCs of healthy mobilized peripheral blood samples. Mock and gene-corrected group: erythroid cells from untreated and gene-edited CD34^+^ HSPCs, respectively.

**
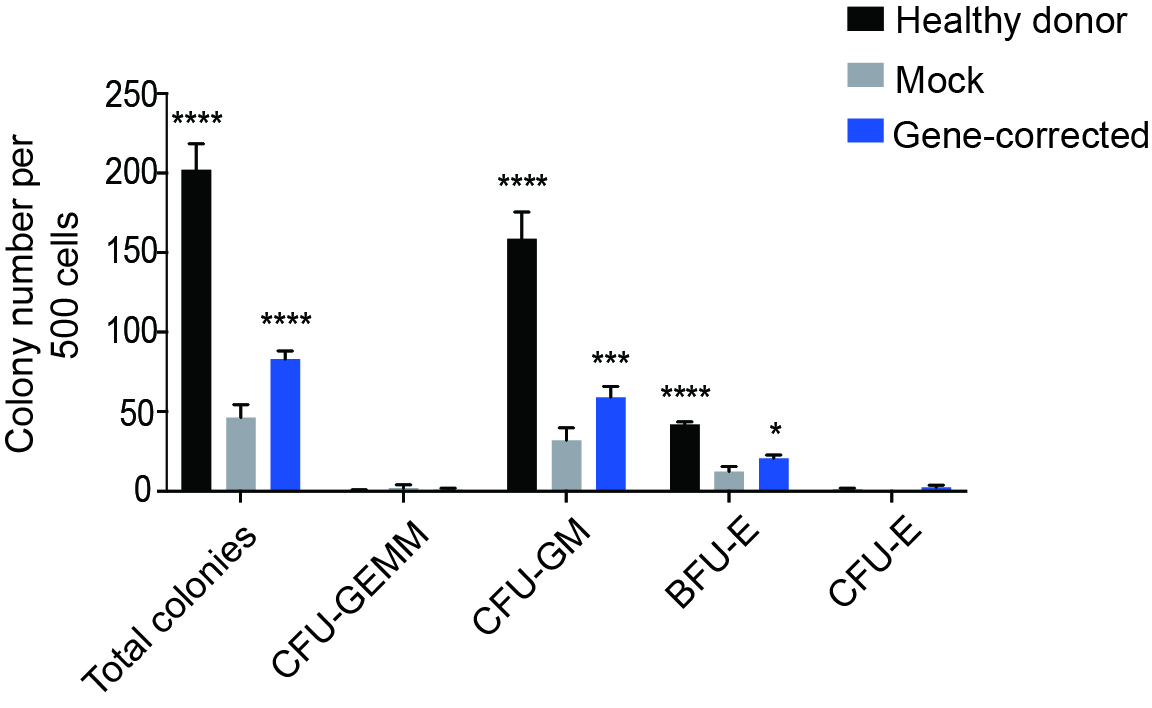
**

**Figure. S6. *In vitro* multi-lineage differentiation analysis by colony-forming unit assay after ALAS2 gene correction.** Human CD34^+^ HSPCs treated with the CRISPR/Cas9 system were examined by CFU assays, and various types of colonies formed by healthy donor cells, by gene-corrected and unedited CD34^+^ HSPCs from XLSA patients were counted. n=3 independent experiments. All data are shown as the mean±SD. *P<0.05, ***P<0.001, ****P<0.0001.


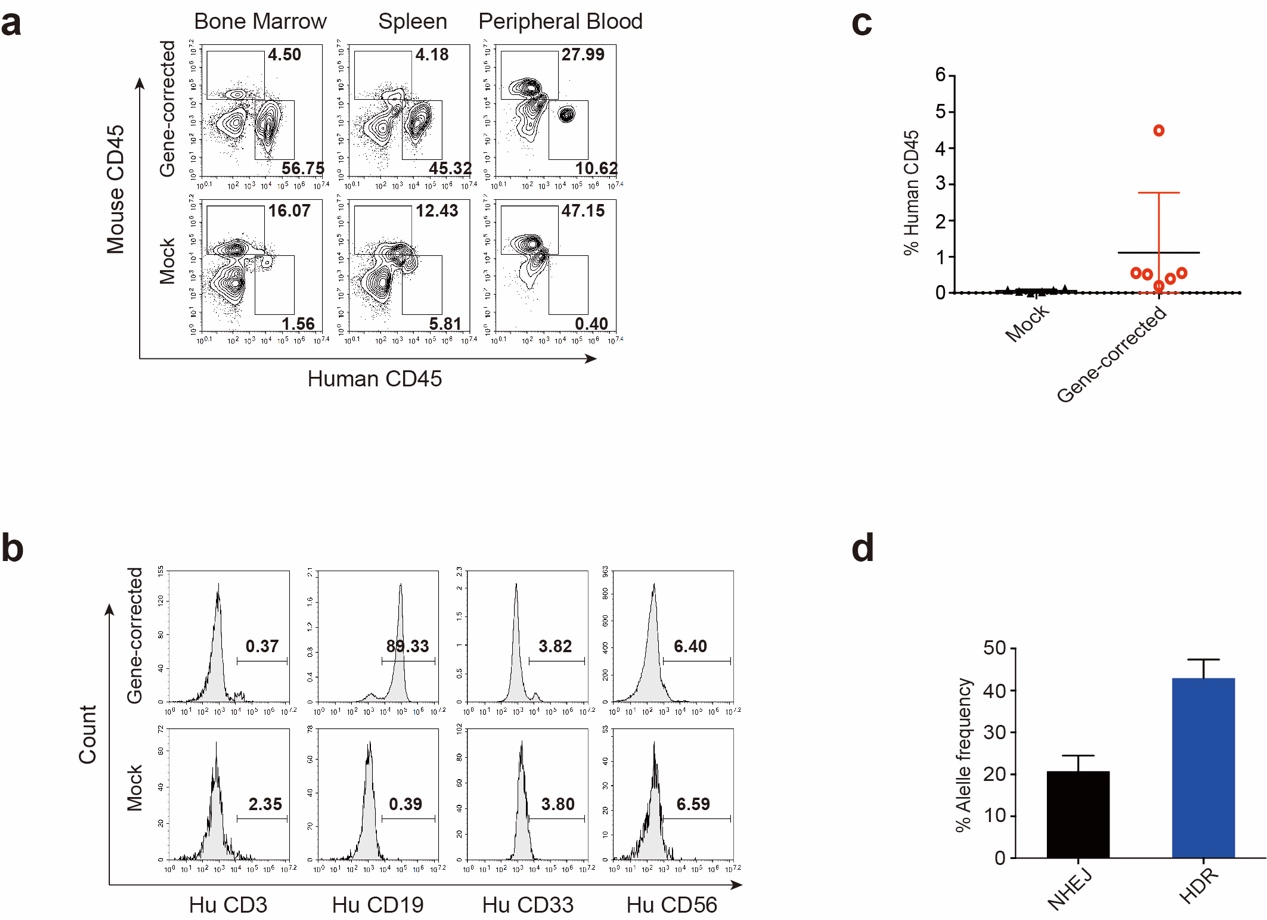


**Figure. S7. *In vivo* assay of CD34^+^ HSPCs from XLSA patients in NPG mice after ALAS2 gene correction.** (a) Representative FACS plots evaluating the percentage of human CD45^+^ cells in the peripheral blood, spleen and bone marrow of reconstituted mice transplanted with unedited mock or gene-edited CD34^+^ HSPCs from XLSA patients. (b) Representative FACS plots from one reconstituted mouse showing the multilineage potential of unedited mock and gene-edited CD34^+^ HSPCs from XLSA patients. T lymphocytes (Human CD3^+^), B lymphocytes (Human CD19^+^) myeloid cells (Human CD33^+^), and NK cells (Human CD56^+^) were detected. (c) The percentages of human CD45^+^ cells were analyzed in the bone marrow of mice 12 weeks after reconstitution by secondary transplantation. n= 6 mice per group. (d) Sanger sequencing analysis showing efficient gene correction (HDR) in human hematopoietic cells from the bone marrow of mice 12 weeks after reconstitution by secondary transplantation. n=6 mice. All data are shown as the mean±SD.

**
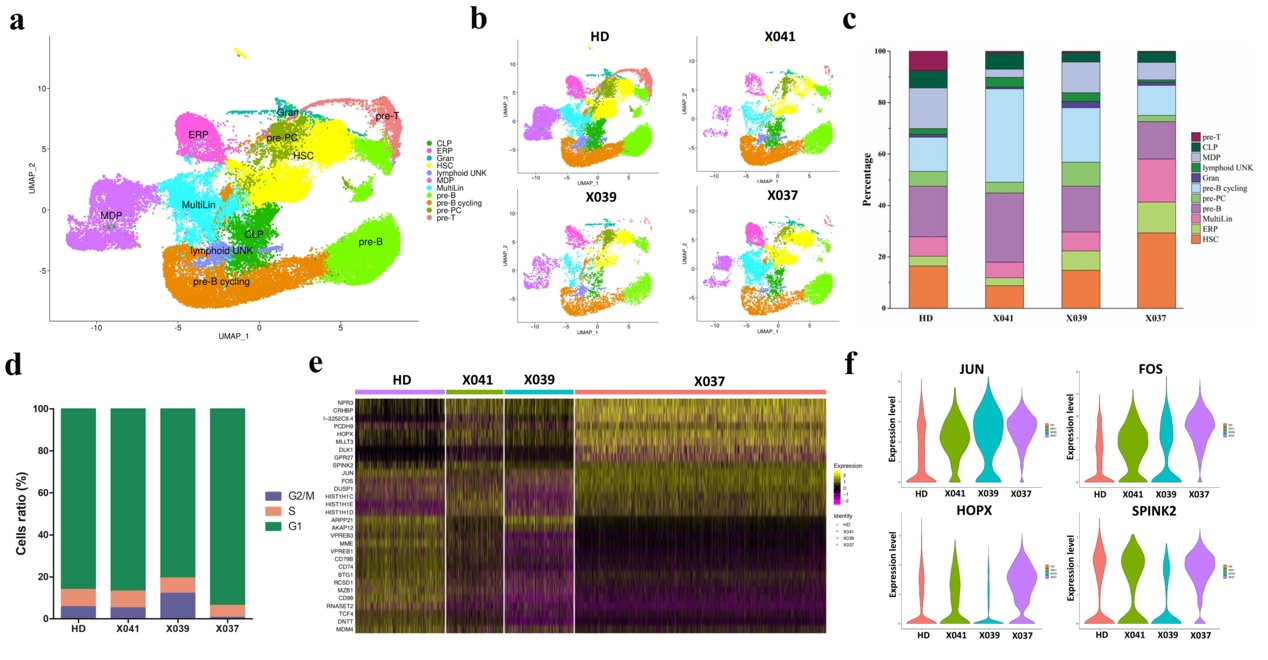
**

**Figure. S8. Single-cell transcriptomic analysis identified heterogeneity of individual XLSA case in hematopoietic maturation dynamics.** (a) UMAP plot by unsupervised clustering showed 11 distinct subsets demarcated by colors in the trajectory of hematopoietic maturation from integrative analysis from three healthy donors (HD) and three XLSA patients. (b) Four UMAP plots show separation of the eleven subsets with discriminated colors from HD and XLSA patients, respectively. (c) Stacked barplots represented subsets compositions across HD and XLSA patients. (d) Stacked barplots showed cell cycle phases distribution of HSC/LMPP cluster across HD and XLSA patients. (e) Heatmap hierarchically displays with top 30 differentially expressed genes in the HSC subset across HD and XLSA patients referred by single cell transcriptomic profiling. (f) Violin plots showed expression levels across HD and XLSA patients with four of the differentially expressed genes (JUN, FOS, HOPX, and SPINK2) enriched from DE analysis in Figure E.

**Table.S1. Hematological characteristics of the investigated pedigree with *ALAS2* intron 1 GATA binding site mutations**

The normal value of each clinical examination is shown in brackets; Hb: hemoglobin; MCV: mean corpuscular volume; MCH: mean corpuscular hemoglobin; RDW: red cell distribution width.

**Table.S2. List for sgRNAs and single-strand oligonucleotide (ssODN) donor sequence**

| sgRNA-1 | AACTCTGGCAACTTTACCTG |
| --- | --- |
| sgRNA-2 | CAACTTTACCTGTGGTCTGC |
| sgRNA-3 | GGGCTGAGCCTGCAGACCAC |
| ssODN | TCCCACGCCCTGGTCTCAGCTTGGGGAGTGGTCAGACCCCAATGGCGATAAACTCTGGCAACTTTATCTGTGGTCTGCAGGCTCAGCCCCAAGTGCT |

**Table.S3. PCR primer sequences for qRT-PCR and other assays**

| *ALAS2*-Forward | CAGTTCCTGTTTGGTATTGGACG |
| --- | --- |
| *ALAS2*-Reverse | TGCCTTCTGCACAATCTTGCT |
| *GATA1*-Forward | CTGTCCCCAATAGTGCTTATGG |
| *GATA1*-Reverse | GAATAGGCTGCTGAATTGAGGG |
| *GAPDH*-Forward | GAAGGTGAAGGTCGGAGTC |
| *GAPDH-*Reverse | GAAGATGGTGATGGGATTTC |

**Table.S4. PCR primer sequences for indels efficiency and off-target profiling analysis**

| Site ID | Sequence | Primer F | Primer R |
| --- | --- | --- | --- |
| On-target | AACTCTGGCAACTTTACCTGTGG | GGCCACTTCACAAGGTAGGT | GCCAGATGCTCAGACGTGAT |
| POT-1 | AACTtTGGCAgCTTTAaCTGTGG | TGTGCCCAGCATCAATAGCA | TGCTGACTCTCCCAGTGTTT |
| POT-2 | AACTCTGagAACaTTACCTGGGG | AACCGTGTCAGCCGAGAAAA | AAGCCAGGCTTCAGGTAGTC |
| POT-3 | tACTCTGGCAttTTTACCTGAGG | TGGTCCATGGCACCATTTGT | TGGCTGGTACTCTGGCATTT |
| POT-4 | cAaTCTGGCAACcTTACCTGGGG | GGGGAGCATGCAAGATGGA | TGTGCTCAATCTGGCAACCT |
| POT-5 | gACTCTGGCAACTcTACCTcTGG | CCATGACTGAATTTCCCACTCGTA | GTTGGGGTTGCCAGGTAAGA |
| POT-6 | AAaTCTGGCAACccTACCTGTGG | AGAGGGTGCCCATCTACACT | ATTCCTGCTTCCCCGTGATG |
| POT-7 | AAaTCTGaCAACTcTACCTGGGG | GGCAATGAGACCCTGTCTGA | AGCATGAAAACGGACTATCACAGT |
| POT-8 | ttCTCTGGtAAgTTTACCTGTGG | AGAAATATCCCCAAATGGTGCAA | AGCCAGTTTCTTTTTCTAACACAGA |
| POT-9 | gAaTCTGGgAACcTTACCTGCAG | CCTCCACTCTCACCGACACT | CCCAGGGCAAATCCAGAAGT |
| POT-10 | AgCTCTGGCtgCTTTACCTtGGG | TGTTAAGCCCTCCCCTCTGA | GTTGCGGTGACATGTGCAAA |
| POT-11 | gACTCTGGaAACTTTACaTaTGG | ACTTCCGTCAGCACACCTTT | AGGCCACAAGTCTCAATACACT |
| POT-12 | AACcaTGGaAACTTTACaTGGGG | CTTGGCCTGGAATGGTCTCT | TGGATAGGGAAGGCCTGACA |
| POT-13 | cAtTCctGCAACTTTACCTGCAG | ATCCAGAGTGCCAGGACTCA | GCAAGGGCAGGATGGTAACT |
| POT-14 | ttCTCTGaCAACcTTACCTGAAG | ACTGGCATGGTCTAAGCCATA | AGCTCCCACCTGATTTTACTTT |
| POT-15 | ttaTCTaGCAACTTTACCTaGGG | AAGCCACATGGGGACTTCAG | AAGCTAGGGTTCCCGGATTT |
| POT-16 | ttCTCTtGCAgCTTTACCTaTGG | TGGCAGTAGAGGCAGGAGAA | AGCAGACTAATTGGGGCCAATA |
| POT-17 | AgaTgTaGCAACTTTACaTGGGG | CCCCCTAAGAGACAGCACAGA | CGGATGATGCTGGCAGAGTA |
| POT-18 | ttCcCTGGCAACcTTACCTaAAG | AGACCCTGCCCTCATAGACT | TCACCTGACATGGGCAACTG |
| POT-19 | gAtaCaGGtAACTTTACCTGCGG | CGGTACAAGCTGCTGGTTCT | CCTTCCCCTTTTCAGCTGAGA |
| POT-20 | tAtgCTGGCAcaTTTACCTGTGG | GGTCCTCCAAGTCAGAGCTT | TGAGAGGCACCCAGAAAAGA |
| POT-21 | gcaTCTGGagACTTTACCTGAGG | GGGTGGGTTTGAGTGGGAAA | CCCCAGAACTCTTGGCTTGA |
| POT-22 | tgCaCTaGCAACTTTACaTGAGG | GGCAACTGGTATTTCCACATCTG | GGGAGCATGGACAGAAGTGT |
| POT-23 | AAaTCTaaCAACTTTACCCaAGG | TGGCAAGTGGCTAGCAAAGT | CCCATGCAATGTTTGGAGCAT |
| POT-24 | ActTtTaGCAACTTaACCTGGGG | CTCCAGCCCAGGCAATAGAA | CTTGTTACTGGCACGTTCCT |
| POT-25 | cctTtTaGCAACTTTACCTGTAG | TGGTGGCATTCAAAAGTTTCAGA | ACCCCCTCTACCCACCTTTT |
| POT-26 | ggCagTaGCAAtTTTACCTGGGG | TCAGCAACCCATCCCTAGTC | TGCTGCCCCTGTTATGTCTT |
| POT-27 | tgacCTaGCAACcTTACCTGAGG | GATGCTGCTTGTCCCCTTCT | AGGTGAGGGTGGAGTGAAGT |
| POT-28 | gtaTCTGGtAtCcTTACCTGAGG | AGAGGCCAGGTGATCTGCTA | CGAACCTCTGTTCCCATGCT |
| POT-29 | AcacCaGGCAACcTTACCTTGGG | CCACCCACACCCCAGATTTT | ACAAACAACTGACCAAGTTCTGA |
| POT-30 | tcaTCTaGtAtCTTTACCTGTGG | AGGCAGCCAAAGTAGAACCT | GAGGTGCCCTCTTTGATCCA |
| POT-31 | tcaTCaGGCAgCTTTACCTtTGG | GTGAAGGTGGCACGTGGATA | GCTTCTGCCTGGGAAAGACA |
| POT-32 | cctTtTGGCAgCcTTACCTGTGG | TAACCCTAGCCACCCACTCA | AGTGCCTGCCATGTGTTAGG |
